# Impact of Distance and Proficiency on Shooting Kinematics in Professional Male Basketball Players

**DOI:** 10.3390/jfmk7040078

**Published:** 2022-09-30

**Authors:** Dimitrije Cabarkapa, Damjana V. Cabarkapa, Nicolas M. Philipp, Drake A. Eserhaut, Gabriel G. Downey, Andrew C. Fry

**Affiliations:** Jayhawk Athletic Performance Laboratory—Wu Tsai Human Performance Alliance, University of Kansas, Lawrence, KS 66045, USA

**Keywords:** sport, coaching, biomechanics, performance, free-throw, two-point, three-point

## Abstract

Shooting efficiency is one of the key performance parameters related to securing the desired game outcome at various levels of basketball competition, and it is largely influenced by the biomechanical adjustments incorporated during the preparatory and release phase of the shooting motion. Thus, the purpose of the present study was twofold: (a) to examine the differences in the kinematic characteristics between free-throw, two-point, and three-point shots, and (b) to examine the differences between shooters with excellent (≥80%) and good (<80%) levels of shooting proficiency. A total of 10 professional male basketball players performed 5 free-throw (4.57 m), two-point (5.18 m), and three-point (6.75 m) shots, combining for a total of 150 shots. A high-definition camera recording at 120 fps was used to capture the shooting motion from a sagittal point of view, and video analysis software was used to analyze the kinematic variables of interest. The findings of the present study reveal that the kinematic characteristics during the preparatory phase of the shooting motion remain unchanged between free-throw and two-point shots. Three-point shots required lower elbow positioning, influenced by greater knee and hip flexion when compared to free-throw and two-point shots. The release angle was notably lower for shots attempted beyond the three-point line but remained unchanged between the free-throw and two-point shooting motions. Release height and vertical displacement were significantly greater for two- and three-point shots when compared to free-throw shots, while no difference was observed between the two- and three-point shots. In addition, no significant differences in shooting kinematics were observed between those participants with excellent and good levels of shooting proficiency.

## 1. Introduction

Basketball is one of the most popular international sports. It is a fast-paced game in which the only way to score is by putting the ball through the basket [1]. This can be achieved by attempting free-throw, two-point, and three-point shots. The efficiency of the aforementioned shooting motions has been shown to be one of the key factors in securing the desired game outcome across various levels of basketball competitions [2,3,4,5]. Therefore, it is of critical importance that each player possesses elementary knowledge regarding the proper shooting form as well as what biomechanical adjustments are needed when increasing the shooting distance.

Previous research has found that proficient free-throw shooters (i.e., ≥70%) tend to have greater flexion in the ankle, knee, and hip joints during the preparatory phase of the shooting motion [6,7]. In an investigation focused on analyzing the differences in the kinematic characteristics between made and missed free-throw shots during a learning process, Ammar et al. [8] found that lower knee flexion during the preparatory phase and greater knee extension during the release phase (i.e., greater total movement) was positively associated with successful shooting outcomes. Also, free-throw shots released at a greater height were found to have an increased probability of success [9,10,11].

When examining the kinematic parameters of the jump shot motion, proficient two-point shooters (i.e., ≥50%) attained greater flexion in the elbow and shoulder joints, while proficient three-point shooters (i.e., ≥40%) kept the torso in a near-vertical position by attaining less hip flexion during the preparatory phase of the shooting motion [12]. Alongside greater elbow flexion during the preparatory phase, Yates [13] found that more successful jump shooters (i.e., 3 m and 6 m) were capable of attaining a greater shoulder flexion at the time point of ball release, which ultimately resulted in a greater release angle. Besides optimizing the release height during the jump shot, Knudson [14] has recommended that shooters should aim to achieve a release angle of between 49–55 degrees above the horizontal plane, although Okazaki & Rodacki [15] have reported greater release angle magnitudes (i.e., ~65 degrees).

While the importance of the previously mentioned kinematic characteristics for the success of a shooting motion remains undisputed, there is a lack of scientific literature focused on studying how they change with an increase in shooting distance. To our knowledge, only a few studies have attempted to address this issue [15,16,17]. Elliott & White [16] found no difference in the ankle, knee, hip, and elbow flexion during the preparatory phase of the shooting motion between two-point and three-point shots (i.e., 4.0 m and 6.25 m). Satern [17] found a decrease in the release angle and an increase in the vertical displacement as the shooter started to move further away from the basket. Moreover, Okazaki & Rodacki [15] found no difference in angular amplitude between short-, mid-, and long-range jump shots, while the release angle significantly decreased with an increase in shooting distance.

Therefore, to bridge a gap in the scientific literature and obtain additional insight into biomechanical parameters of elementary shooting motions, the purpose of the present study was twofold: (a) to examine the differences in the kinematic characteristics between free-throw, two-point, and three-point shots, and (b) to examine the differences in the kinematic characteristics between shooters with excellent and good levels of shooting proficiency.

## 2. Materials and Methods

### 2.1. Participants

A total of 10 professional male basketball players (age = 25.7 ± 2.5 years, height = 191.8 ± 11.5 cm, and body weight = 88.7 ± 12.1 kg) with previous collegiate playing experience that were under professional contract or between contracts at the time of the data collection volunteered to participate in the present study (e.g., France ProA, Germany ProA). All players were right-hand dominant shooters and were free of musculoskeletal injuries that would limit full joint range motion. All testing procedures performed in this study were previously approved by the University’s Institutional Review Board, and all participants signed an informed consent document.

### 2.2. Procedures

The testing procedures performed in the present study resembled the methodology developed by Cabarkapa et al. [12]. Upon arrival at the basketball gym, participants performed a standardized warm-up procedure, composed of dynamic stretching exercises (e.g., high knees, butt-kicks, lunge-and-twist, A-skips, karaoke, pogo jumps) and 15 min of partner shooting (i.e., one player rebounds the ball while the other one shoots, alternating roles for every 10 shooting attempts). After the completion of the warm-up protocol, each participant attempted five free-throw (4.57 m), two-point (5.18 m), and three-point (6.75 m) shots, combining a total of 150 shots across all participants. All shots were attempted from mid-court, directly facing the basket, immediately after receiving the ball from the passer. Each shot and set of five free-throw, two-point, and three-point shots were separated by 5–10 s and 1 min rest interval, respectively. A high-definition camera (Sony Cyber-Shot RX10 IV, Tokyo, Japan) recording at 120 fps was used to capture the shooting motions, and video analysis software (Kinovea, Version 0.9.5) was used to analyze the kinematic variables of interest. The camera was positioned 10 m away, perpendicular to the shooting plane of motion (i.e., sagittal view), respective to the shooting location (i.e., free-throw, two-point, and three-point). To minimize distraction and possible influences of fatigue, participants individually performed the testing procedures, and research assistants were present to complete rebounding and passing tasks. The goal height (3.05 m) and basketball size (0.75 m) corresponded to men’s basketball international regulation standards.

### 2.3. Variables

The following kinematic variables were obtained during the preparatory phase of the shooting motion, defined as the timepoint of the initial concentric motion while the shooter was still on the ground: *knee angle* (i.e., internal angle between the thigh and shank), *hip angle* (i.e., internal angle between the torso and the thigh), *ankle angle* (i.e., relative angle between the shank and the ground), *elbow angle* (i.e., internal angle between the upper arm and forearm), *shoulder angle* (i.e., relative angle between the upper arm and torso), and *elbow height* (i.e., perpendicular distance between the olecranon process and the ground divided by the participant’s height).

**Figure 1:**
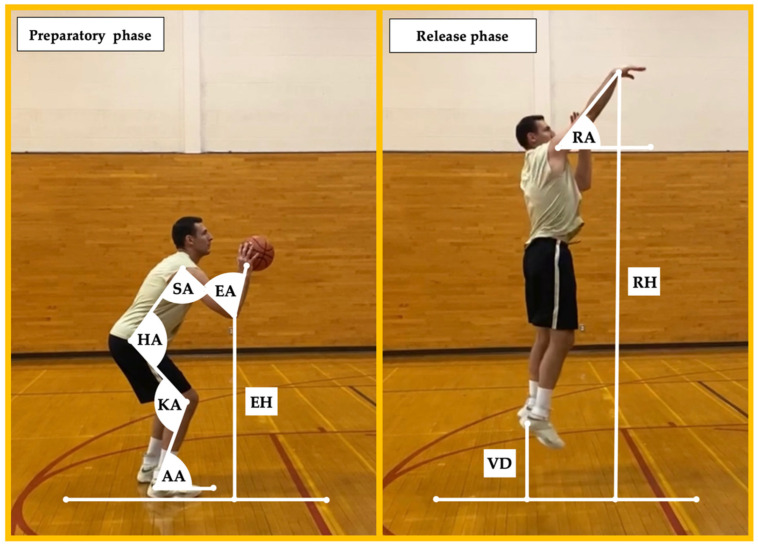
Graphical representation of kinematic variables examined in the present study. KA = knee angle, HA = hip angle, AA = ankle angle, EA = elbow angle, SA = shoulder angle, EH = elbow height, RA = release angle, RH = release height, and VD = vertical displacement.

The following kinematic variables were obtained during the release phase of the shooting motion, defined as the timepoint when the shooter lost contact with the ball: *release angle* (i.e., relative angle between the fully extended upper limb and a line parallel to the ground), *release height* (i.e., perpendicular distance between the hand and the ground divided by the participant’s height), and *vertical displacement* (i.e., perpendicular distance between the posterior calcaneus and the ground). The detailed graphical representation of kinematic variables examined in the present investigation is presented in Figure 1.

### 2.4. Statistical Analysis

Descriptive statistics, means, and standard deviations ($\bar{x}$ ± SD) were calculated for each dependent variable. A repeated measures MANOVA was used to examine the statistically significant differences in the dependent variables between the three types of shooting motions (i.e., free-throw, two-point, and three-point). Follow-up ANOVAs, with Bonferroni post hoc adjustments, were used to examine the statistically significant main effects where needed. If the assumption of sphericity was violated, a Greenhouse–Geisser correction was used. In addition, independent *t*-tests were used to examine statistically significant differences in the dependent variables between the participants with excellent (i.e., made ≥4 shots/≥80%; n = 6) and good levels (i.e., made <4 shots/<80%; n = 4) of shooting proficiency, separately for each shooting motion. Statistical significance was set *a priori* to *p* ≤ 0.05. All statistical analyses were completed with SPSS (Version 27.0; IBM Corp., Armonk, NY, USA).

## 3. Results

The average free-throw, two-point, and three-point shooting accuracy across all players was 76.0 ± 24.6%, 68.0 ± 25.3%, and 64.0 ± 22.7%, respectively. The overall MANOVA model was statistically significant (F = 7.724; *p* < 0.001), indicating notable differences in the biomechanical characteristics between the three types of shooting motions examined in the present study. Statistically significant differences were present for knee angle (F = 6.424, *p* = 0.025), hip angle (F = 7.116, *p* = 0.005), elbow height (F = 8.014, *p* = 0.011), release angle (F = 13.897, *p* = 0.003), release height (F = 9.611, *p* = 0.001), and vertical displacement (F = 42.796, *p* < 0.001). No significant differences were observed for ankle angle (F = 1.223, *p* = 0.305), elbow angle (F = 0.563, *p* = 0.579), and shoulder angle (F = 2.286, *p* = 0.162).

Significant differences between the free-throw and two-point shooting motions were found for the release height (*p* = 0.005) and vertical displacement (*p* < 0.001). Significant differences between the free-throw and three-point shooting motion were found for the knee angle (*p* = 0.048), hip angle (*p* = 0.042), elbow height (*p* = 0.044), release angle (*p* = 0.012), release height (*p* = 0.050), and vertical displacement (*p* < 0.001). Lastly, significant differences between the two-point and three-point shooting motion were found for the knee angle (*p* = 0.006), hip angle (*p* = 0.011), elbow height (*p* = 0.045), and release angle (*p* = 0.005) (see Table 1).

The average free-throw, two-point, and three-point shooting accuracy for the excellent shooters was 93.3 ± 6.3%, 88.0 ± 10.9%, and 85.0 ± 10.0%, and for good shooters, it was 50.0 ± 11.5%, 48.0 ± 17.9%, and 50.0 ± 16.7%, respectively. No statistically significant differences in any of the dependent variables examined in the present study were observed between the excellent and good shooters (see Table 2).

## 4. Discussion

### 4.1. Preparatory Phase

The findings of the present study reveal that the kinematic characteristics during the preparatory phase of the shooting motion remain unchanged between the free-throw and two-point shots. Three-point shots required lower elbow positioning and greater flexion in the knee and hip joints when compared to the free-throw and two-point shots. Also, it is important to note that the lower elbow positioning did not result from less flexion in the shoulder joint, as no significant differences in shoulder angle were observed, but rather occurred as a consequence of greater knee and hip flexion.

When examining the changes in the biomechanical characteristics of the shooting motion in male basketball players, Okazaki & Rodacki [15] found no significant differences in the ankle, knee, and hip joint angular displacement and amplitude with an increase in shooting distance (i.e., 2.8 m, 4.6 m, and 6.4 m). Similar observations were made by Elliot & White [16] when studying female basketball players. Ankle, knee, hip, and elbow joint angles remained unchanged during the preparatory phase of the shooting motion between the two- and three-point shots (i.e., 4.0 m and 6.25 m) [16]. In addition, Miller & Bartlett [18] found that trunk angle (i.e., the relative angle between the torso and the ground) in male basketball players tends to remain consistent with an increase in shooting distance (i.e., 2.7 m, 4.6 m, and 6.4 m) regardless of the playing position (i.e., center, forward, or guard). The previously mentioned research reports align with the findings of the present study, where no significant differences in kinematic characteristics during the preparatory phase of the shooting motion were observed between the free-throw and two-point shots (i.e., 4.57 m and 5.18 m). Yet, the same reports seem to be contradictory to the results pertaining to the shots attempted beyond the three-point line (i.e., 6.75 m). This discrepancy may be attributed to differences in the testing methodologies, primarily due to the distance from which the long-range shots were attempted (e.g., 6.4 m vs. 6.75 m).

If portrayed as a dynamic system, adjustments in shooting form are likely to occur as the difficulty of the task increases, such as moving further away from the basket, while also being accompanied by a decrease in shooting accuracy [19]. While further research is warranted to examine how kinetic characteristics change as a result of an increase in shooting distance, we can assume that greater flexion in the knee and hip joints, accompanied by lower elbow positioning, were the kinematic adjustments made to shooting form that were used to generate more force needed to propel the ball towards the basket and compensate for an increase in shooting distance. In addition, the magnitudes of kinematic variables examined in the present study during the preparatory phase of the shooting motion are similar to those values previously reported in proficient male basketball shooters, which is expected considering the participant’s level of expertise (i.e., professional basketball players) [6,7,12,20].

### 4.2. Release Phase

Alongside alterations in shooting form during the preparatory phase, significant differences in the kinematic characteristics during the release phase of the shooting motion were observed in the present study. The release angle was notably lower for shots attempted beyond the three-point line but remained unchanged between the free-throw and two-point shooting motions. Also, release height and vertical displacement were significantly greater for two- and three-point shots when compared to free-throw shots, while no difference was observed amongst them.

Previous research has found that release angle is positively associated with the entry angle of the ball at the rim as one of the key factors determining the success of the shooting motion [1,15,18]. A greater release angle would allow the shooter to use a greater width of the basket, which could ultimately optimize shooting accuracy [1]. However, as the shooter moves away from the basket and starts attempting mid- and long-range shots, the release angle should decrease (i.e., less shoulder flexion) [21]. When examining the shots projected from various distances (i.e., 3.0 m to 8.2 m), Satern [17] found a gradual decrease in release angle with an increase in shooting distance in collegiate male athletes. Likewise, Elliott & White [16] found significantly lower release angles for three-point shots (compared with two-point shots) in female basketball players (i.e., 4.0 m and 6.25 m), which is in agreement with the results obtained in the present investigation. Nevertheless, despite being lower for two-point shots, the mean values of release angle did not reach a level of statistical significance. This may be attributed to the close proximity of the sites from which these two types of shooting motions were attempted (i.e., 4.57 m and 5.18 m) and/or the pure nature of the shooting motion (i.e., stationary vs. jump shot).

Additionally, simultaneous to release angle, release height has been shown to be of critical importance for the success of the shooting motion as it allows for a larger margin of error [1,9,14,22]. Thus, instructing a shooter to jump as close to vertical as possible and release the ball at the top of the jump are some of the key coaching cues that should be incorporated when teaching mid- and long-range jump shots [14]. Based on the findings of the present study, we can conclude that the participants successfully implemented the aforementioned coaching strategies. Vertical displacement was significantly greater for the two- and three-point shots when compared to the free-throw shots, which ultimately resulted in a greater release height. Interestingly, no significant differences in vertical displacement and release height were detected between the two- and three-point shooting motions. Similar observations were made by Okazaki & Rodacki [15] when examining the difference in maximal vertical displacement between mid- and long-range jump shots (i.e., 4.6 m and 6.4 m). In addition, all the values obtained for the kinematic parameters during the release phase of the free-throw, two-point, and three-point shooting motions were similar to the previous research reports focused on examining proficient male basketball players. As previously noted, this is expected based on the participants’ playing expertise (i.e., professional male basketball players) [6,12,20].

### 4.3. Shooting Proficiency

While several kinematic adjustments in shooting form occurred as the shooting distance increased, no significant differences were observed between those participants with excellent and good levels of shooting proficiency. All kinematic variables of interest examined in the present study for each type of shooting motion (i.e., free-throw, two-point, three-point) were similar. Previous research has observed prominent differences in multiple biomechanical parameters when comparing proficient to non-proficient free-throw, two-point, and three-point shooters [6,7,12]. Proficient free-throw shooters (i.e., ≥70%) demonstrated greater flexion in the ankle, knee, and hip joints during the preparatory phase of the shooting motion and greater release height at the timepoint of the ball release when compared to non-proficient shooters (i.e., <70%) [6]. Higher elbow placement and greater elbow flexion during the preparatory phase of the shooting motion were characteristic of proficient two-point shooters (i.e., ≥50%) [12]. Moreover, proficient three-point shooters (i.e., ≥40%) attained a greater release height and vertical displacement during the release phase of the shooting motion [12].

Being unable to observe any significant differences between excellent and good shooters may be predominantly attributed to the cohort of participants examined in the present study. The participants were professional male basketball players with an extensive amount of playing experience. Therefore, it is reasonable to assume that they already possessed elementary knowledge regarding the proper shooting form and had already incorporated the kinematic adjustments observed in previous research reports. Concurrently, these findings imply that there are other biomechanical parameters that affected the successful execution of the free-throw, two-point, and three-point shooting motion, which have not been examined in the present study. Some of them may be related to joint angular velocities (e.g., elbow, wrist, knee), as well as coordination-variability patterns, and warrant further investigation [1,8,23]. Additionally, while video analysis remains a practical and affordable method for the assessment of shooting form, it may lack the ability to obtain the aforementioned biomechanical variables of interest. Thus, a possible solution to this problem may be found in the usage of innovative markerless motion capture systems that allow for the non-invasive assessment of sport-specific skills [24,25,26,27].

### 4.4. Limitations

While these findings offer additional insight into the kinematic characteristics of the shooting motions of professional male basketball players and how they change with an increase in shooting distance, the present study is not without limitations. The overall sample size, as well as the number of attempted shots for each shooting motion (i.e., free-throw, two-point, three-point), could have been larger. Also, the testing procedures were conducted without the presence of a defender and under non-fatiguing conditions, which do not directly resemble on-court basketball playing demands and should be studied in the future.

### 4.5. Practical Application

Knowing which biomechanical adjustments (in shooting form) need to be incorporated when transitioning from mid- to long-range shots can help basketball coaches and players optimize the learning process and can ultimately lead to improvements in shooting accuracy. Instructing players to lower their elbow positioning by flexing their knees and hips during the preparatory phase of the shooting motion and jumping and releasing the ball at the highest point of their jump may be beneficial coaching cues given to players that can potentially lead to improvements in three-point shooting performance.

## 5. Conclusions

While no significant differences in the kinematic characteristics were observed between free-throw and two-point shots during the preparatory phase of the shooting motion, the three-point shots required lower elbow positioning, primarily resulting from greater flexion in the knee and hip joints. Greater release height and lower release angles, accompanied by greater vertical displacement, were observed for three-point shots when compared to free-throw and two-point shots. The release height and vertical displacement were significantly greater for two- and three-point shots when compared to free-throw shots, while no differences were observed amongst them. In addition, no significant differences in the kinematic variables examined in the present study were observed between those participants with excellent and good levels of shooting proficiency, which implies that there might be other biomechanical parameters influencing the success of the shooting motion that warrant further research.

## Figures and Tables

**Table 1:** Descriptive statistics, means and standard deviations ($\bar{x}$ ± SD), for each dependent variable observed for free-throw, two-point, and three-point shooting motions.

	**Knee angle (deg)**	**Hip angle (deg)**	**Ankle angle (deg)**
Free-throw	121.5 ± 8.8 *	143.9 ± 8.4 *	61.2 ± 6.7
Two-point	116.7 ± 7.4 *	141.1 ± 8.1 *	61.5 ± 5.3
Three-point	112.5 ± 7.4	135.5 ± 8.4	58.5 ± 4.7
	**Elbow angle (deg)**	**Shoulder angle (deg)**	**Elbow height (ratio)**
Free-throw	61.9 ± 13.7	78.9 ± 26.9	0.701 ± 0.085 *
Two-point	58.2 ± 16.6	72.7 ± 26.4	0.676 ± 0.091 *
Three-point	63.6 ± 21.6	65.8 ± 31.4	0.619 ± 0.092
	**Release angle (deg)**	**Release height (ratio)**	**Vertical displacement (cm)**
Free-throw	60.8 ± 6.3 *	1.307 ± 0.067	15.3 ± 5.1
Two-point	58.9 ± 7.4 *	1.378 ± 0.073 ^†^	26.9 ± 5.6 ^†^
Three-point	56.9 ± 8.5	1.377 ± 0.093 ^†^	31.2 ± 7.3 ^†^

Note: * significantly different when compared to three-point shooting motion. ^†^ Significantly different when compared to free-throw shooting motion; (*p* < 0.05).

**Table 2:** Descriptive statistics, means and standard deviations ($\bar{x}$ ± SD), for each dependent variable observed for excellent (≥80%; n = 6) and good (<80%; n = 4) free-throw, two-point, and three-point shooters.

**Free-throw**	**Excellent shooters**	**Good shooters**
Knee angle (deg)	123.1 ± 9.3	119.1 ± 8.8
Hip angle (deg)	145.5 ± 7.3	141.6 ± 10.6
Ankle angle (deg)	62.8 ± 6.5	58.9 ± 7.2
Elbow angle (deg)	57.8 ± 12.9	68.2 ± 14.1
Shoulder angle (deg)	74.6 ± 26.1	85.3 ± 31.0
Elbow height (ratio)	0.692 ± 0.084	0.713 ± 0.098
Release angle (deg)	60.1 ± 5.6	61.8 ± 7.9
Release height (ratio)	1.301 ± 0.078	1.316 ± 0.055
Vertical displacement (cm)	14.2 ± 4.7	16.9 ± 5.9
**Two-point**	**Excellent shooters**	**Good shooters**
Knee angle (deg)	117.7 ± 6.9	115.7 ± 8.6
Hip angle (deg)	141.8 ± 4.2	140.5 ± 11.4
Ankle angle (deg)	63.2 ± 6.1	59.7 ± 4.2
Elbow angle (deg)	62.3 ± 22.8	54.2 ± 7.9
Shoulder angle (deg)	65.7 ± 32.5	79.6 ± 19.8
Elbow height (ratio)	0.658 ± 0.102	0.694 ± 0.085
Release angle (deg)	58.0 ± 7.8	59.8 ± 7.7
Release height (ratio)	1.388 ± 0.092	1.369 ± 0.059
Vertical displacement (cm)	27.2 ± 7.3	26.6 ± 4.1
**Three-point**	**Excellent shooters**	**Good shooters**
Knee angle (deg)	107.9 ± 10.1	115.5 ± 3.4
Hip angle (deg)	135.5 ± 9.1	135.6 ± 8.7
Ankle angle (deg)	57.3 ± 6.6	59.2 ± 3.4
Elbow angle (deg)	55.2 ± 19.7	69.1 ± 22.7
Shoulder angle (deg)	74.9 ± 35.5	59.7 ± 30.1
Elbow height (ratio)	0.622 ± 0.088	0.618 ± 0.103
Release angle (deg)	54.5 ± 9.5	58.6 ± 8.2
Release height (ratio)	1.364 ± 0.089	1.386 ± 0.102
Vertical displacement (cm)	32.6 ± 8.9	30.3 ± 6.8

## Data Availability

The data presented in this study are available on request from the corresponding author.
